# Assessing the Significance of the Circadian Time of Administration on the Effectiveness and Tolerability of OnabotulinumtoxinA for Chronic Migraine Prophylaxis

**DOI:** 10.3390/toxins14050296

**Published:** 2022-04-21

**Authors:** Emmanouil V. Dermitzakis, Michail Vikelis, George S. Vlachos, Andreas A. Argyriou

**Affiliations:** 1 Department of Neurology, “Geniki Kliniki” Euromedica, 54645 Thessaloniki, Greece; manolis.dermitzakis@gmail.com; 2 Headache Clinic, Mediterraneo Hospital, 16675 Glyfada, Greece; mvikelis@headaches.gr (M.V.); gvlachos@neuromed.gr (G.S.V.); 3 Glyfada Headache Clinic, 16675 Glyfada, Greece; 4 Neurology Department of the “Agios Andreas” State General Hospital of Patras, Headache Outpatient Clinic, 26335 Patras, Greece

**Keywords:** OnabotulinumtoxinA, chronic migraine, efficacy, tolerability/safety, circadian time

## Abstract

We aimed to provide insights on the role of the circadian time of administration in influencing the efficacy and tolerability/safety profile of OnabotulinumtoxinA (BoNTA) for chronic migraine (CM) prophylaxis. Methods: We retrospectively reviewed the medical files of BoNTA-naïve patients with CM who completed three consecutive cycles of treatment, according to the standard PREEMPT paradigm. Participants were classified to those scheduled to be treated in the morning hours from 8:00 to 12:00 (AM) or afternoon hours from 13:00 to 18:00 (PM). We then assessed and compared between groups the changes from baseline (T0—trimester before BoNTA’s first administration) to the period after its third administration (T3) in the following efficacy outcomes: (i) mean number of headache days/month, (ii) mean number of days/month with peak headache intensity of >4/10, (iii) mean number of days/month with consumption of any abortive treatment. Safety–tolerability was also compared between groups. Results: A total of 50 AM and 50 PM-treated patients were evaluated. The within-group analysis in both groups showed a significant decrease in all efficacy variables between T0 and T3. However, the between-group comparisons of all BoNTA-related efficacy outcomes at T3 vs. T0 documented comparable improvements between AM vs. PM-treated patients. Safety/tolerability was also similar between groups. Conclusions: We were not able to identify significant differences between patients treated in the AM vs. PM, so as to demonstrate that the circadian time of administration should be considered before initiating BoNTA in CM patients.

## 1. Introduction

Migraine, clinically divided to either episodic or chronic forms, ranks among the most common primary headaches and according to estimates it has a global prevalence of 15.3% [1]. Sufferers from chronic migraine (CM) are those experiencing headaches occurring on 15 days or more per month, with at least 8 of these being typical migraine days, for more than 3 months [2].

CM, despite being much less common than an episodic migraine (EM), accounts for about 2.5% of migraineurs, and carries much higher functional consequences with reduced productivity at work/school and increased rates of pain-related comorbidities, compared to EM, which, as a result of longer average duration of headache, has a greater pain intensity, more severe pain-associated autonomic symptoms, and increased rates of medication overuse headache (MOH) [3,4]. As such, there is an obvious clinical need to commence effective preventative pharmacological therapies to CM patients in order to decrease the monthly frequency and severity of migraine attacks, but also to diminish the need of analgesics consumption.

OnabotulinumtoxinA (BoNTA) was approved to prevent headaches in adult patients with CM, based on the outcomes of two double-blind, randomized, placebo-controlled phases of the PREEMPT clinical program (Phase III REsearch Evaluating Migraine Prophylaxis Therapy) that tested the efficacy/safety of BoNTA and found significant improvements in all efficacy endpoints with BoNTA, compared to placebo, coupled with considerable tolerance and few adverse events (AEs), mainly including neck pain, muscle weakness, and eyelid ptosis [5]. Hence, the pooled analysis of PREEMPT trials demonstrated a favourable benefit–risk ratio of BoNTA.

The mode of BoNTA action in CM prophylaxis is mainly based on indirect inhibition of central sensitization through suppression of neurogenic inflammation and peripheral sensitization. To achieve this, BoNTA is able to evoke a blockage of synaptic vesicle fusion through cleavage of the soluble N-ethylmaleimide-sensitive fusion attachment protein (SNAP-25), so as to inhibit the soluble N-ethylmaleimide-sensitive factor attachment protein receptor (SNARE) complex and achieve the reduction of various pain-modulating neurotransmitters and neuropeptides release from the sensory nerve terminals, including glutamate, calcitonin gene-related peptide (CGRP), and substance P [6].

It was previously demonstrated that CM sufferers with overactivated peripheral trigeminal endings during attacks, characterized by pericranial allodynia and muscle tenderness, unilateral pain in the ophthalmic branch of the trigeminal nerve and also a presence of cranial autonomic symptoms, respond better to BoNTA than others without these phenotypes [7,8]. Moreover, there is also evidence to suggest that the presence of ocular and imploding rather than exploding pain may also predict BoNTA responsiveness [9].

Theoretically, it might be interesting to assess if the circadian time of administration should be considered when scheduling initiation of BoNTA therapy, based on the facts that BoNTA is able to inhibit SNAP-25 for about 4 h (biological half-life) after its administration [9], coupled with evidence demonstrating the fluctuating levels of SNAP-25, according to circadian rhythms [10]. Nonetheless, the role of the circadian time of administration and its potential impact on the efficacy and safety/tolerability profile of BoNTA injections for the prevention of migraines has been thus far minimally explored. Packard et al. [11] were the first to clinically evaluate the significance of the circadian time of administration upon the effectiveness of BoNTA in CM prophylaxis, while the same group also assessed whether the same parameter can influence the safety/tolerability profile of BoNTA [12]. The results of both the latter publications showed that scheduling diurnally active CM patients for afternoon BoNTA injections appear to improve the effectiveness and safety/tolerability profile of BTA therapy, as well as patients’ satisfaction to treatment [11,12]. In our knowledge, there are no additional data to further test the latter assumption.

We have previously reported significant improvement in all clinical efficacy measures, including reduction of mean monthly headache days, days/month with moderate/severe headache intensity, as well as monthly days with intake of abortive therapies after commencing three treatment cycles of BoNTA, compared to baseline [13]. Moreover, we have subsequently demonstrated sustained BoNTA safety/efficacy and tolerability profile in CM patients after the completion of 3–5 years of treatment [14,15]. We herein report the outcome of a retrospective, multi-center study that sought to ascertain if indeed the circadian time of administration can influence the efficacy and tolerability/safety profile of BoNTA for CM prophylaxis, as previously suggested [11,12].

## 2. Results

The medical files of 100 consecutive patients who achieved treatment with the third BoNTA course, according to the study plan, were reviewed. The study sample consisted of 11 males (11%) and 89 females (89%) with a mean age of 43.6 ± 19.3 (range: 21–60) years. Participants were equally treated with BoNTA at an AM, from 8:00 to 12:00 (*n* = 50), vs. PM, from 13:00 to 18:00 (*n* = 50), time of administration. Table 1 describes in detail the baseline epidemiological and clinical characteristics of participants, according to circadian time of BoNTA administration.

### 2.1. Within Group Comparison of Efficacy Headache Outcomes According to BoNTA Time of Administration

#### 2.1.1. AM-Treated Patients (*n* = 50)

The analysis of efficacy variables in this subgroup showed that there was a significant decrease in mean monthly headache days between T0 and T3 (20.8 ± 5.1 vs. 9.3 ± 4.8; *p* < 0.001). In addition, a significant decrease in the number of days/month with peak headache intensity of more than 4/10 (moderate/severe pain) was noted between T0 and T3 (12.4 ± 5.9 vs. 7.7 ± 4.7; *p* < 0.001). Finally, the change in days using acute headache medications per month between T0 and T3 was also significant (16.7 ± 5.6 vs. 7.4 ± 4.3; *p* < 0.001).

A total of 34/50 (68%) patients have successfully achieved response either at 50% (*n* = 16) or at 75% (*n* = 18) at T3 compared to T0, whereas 16 patients (32%) failed to respond to BoNTA (response less than 50%). The efficacy to therapy obviously influenced the BoNTA-attributed perception of change and satisfaction of AM-treated patients as 34 of them remained satisfied to score ≥5 on “Patient Global Impression of Change” (PGIC); specifically, 13 scored 5, 15 scored 6 and 6 scored 7 at PGIC.

#### 2.1.2. PM-Treated Patients (*n* = 50)

The mean monthly headache days were significantly decreased between T0 and T3 follow-ups (18.2 ± 4.1 vs. 6.2 ± 3.5; *p* < 0.001). Moreover, there was a significant decrease in the number of monthly days with peak headache intensity of ≥5 between T0 and T3 (10.4 ± 4.2 vs. 6.2 ± 3.5; *p* < 0.001), while the monthly days with intake of acute headache medications was also significant between T0 and T3 (12.9 ± 6.4 vs. 5.3 ± 3.2; *p* < 0.001).

In total, 38 of 50 (76%) patients were classified as responders. A total of 8 of these responders achieved a 50% reduction and 30 patients obtained a 75% decrease in mean monthly headache days. In total, 12 patients (24%) were considered as non-responders experiencing less than 50% reduction in mean headache days between T0 vs. T3. Among PM-treated patients, 38 scored ≥ 5 on PGIC; specifically, 9 scored 5, 21 scored 6 and 8 scored 7 at PGIC to express their satisfaction to BoNTA therapy.

### 2.2. Between Group Comparison of Efficacy Headache Outcomes According to BoNTA Time of Administration (AM vs. PM)

**Figure 1:**
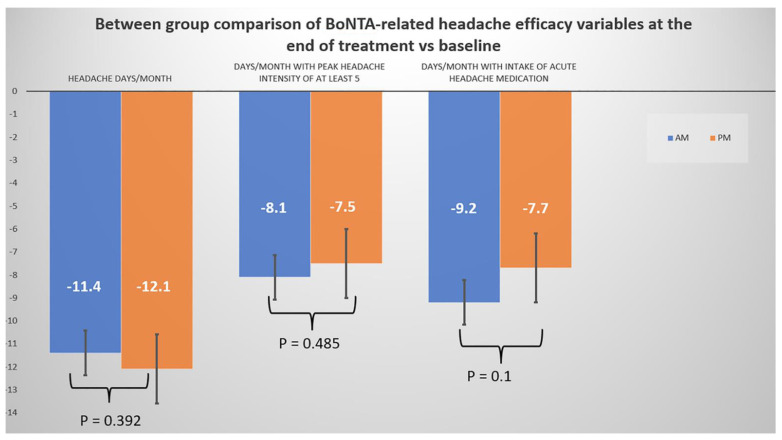
Changes in mean BoNTA-related headache efficacy scores from baseline to the last follow-up (after three BoNTA courses) between treatment groups. Hyphen (-) in Figure 1 refers to minus sign.

Between group comparisons of all BoNTA-related efficacy headache outcomes at the end of treatment vs. baseline demonstrated comparable improvements occurring in both BoNTA AM and PM-treated patients (Figure 1).

**Figure 2:**
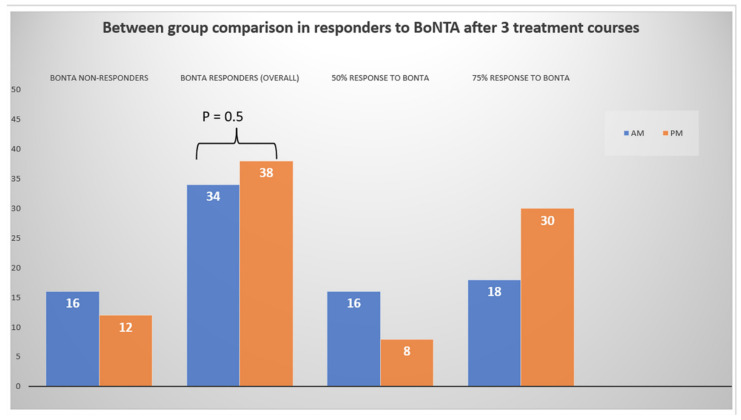
Differences in the number of BoNTA responders at the last follow-up (after three BoNTA courses) between treatment groups.

In addition, as earlier described, the incidence of at least a 50% response to BoNTA therapy was comparable (*p* = 0.5) between AM vs. PM-treated patients (Figure 2).

**Figure 3:**
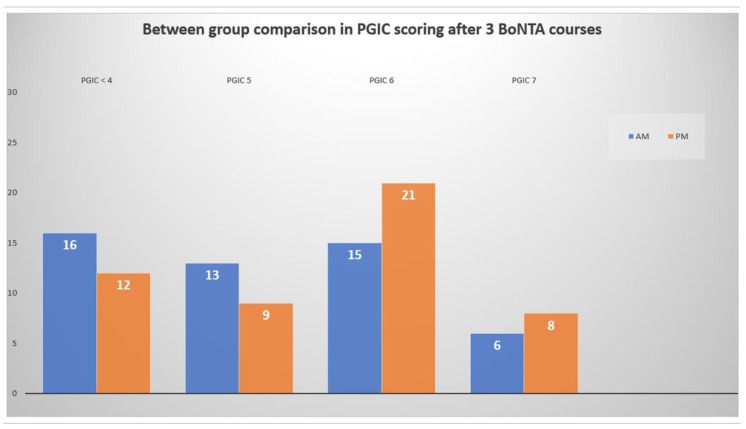
Differences in the number of patients scoring ≥ 5 in “Patient Global Impression of Change” (PGIC) at the last follow-up (after three BoNTA courses) between treatment groups.

Concerning the safety/tolerability to BoNTA therapy, we registered mainly mild and transient side effects at comparable rates between AM vs. PM-treated patients, including shoulder and/or neck pain in six patients (6%), wheals in the injection site in five (5%), ptosis in three (3%), and eyebrow elevation in two patients (2%). Early discontinuation before T3 was noted in just two cases due to neck pain of moderate intensity, equally distributed among treatment groups (1 vs. 1). Overall, as pre-mentioned, a similar number of AM vs. PM-treated patients scored PGIC ≥ 5 to express their satisfaction to BoNTA therapy, because they experienced at least a clinically meaningful benefit, while BoNTA proved safe and well-tolerated (Figure 3).

## 3. Discussion

Chronotherapeutics refer to the identification of the optimal circadian time of administration for maximizing the efficacy and safety/tolerability of a given medication [16]. Considering that both the effectiveness and tolerability of about 500 medications significantly varies, according to circadian scheduling [17], it has been previously demonstrated that a chronotherapeutic approach can be implemented in the treatment of neuropsychiatric and neurodegenerative diseases [18,19,20]. Nonetheless, very few data are available, thus far, to the effect of circadian time of administration upon the efficacy and adverse event profile of BoNTA for preventing CM. Based on evidence suggesting that a diurnal variation in migraine attacks and other inflammatory pain models might occur [21,22,23], only quite few previous studies, conducted by the same group, have demonstrated that both the efficacy and safety/tolerability profile of BoNTA in CM prophylaxis are optimized when diurnally active patients are treated at afternoon rather than at morning hours [11,12].

In the current setting, we sought to replicate the latter findings by assessing in a similar gender and age variation cohort of CM patients, if indeed the circadian timing of injecting BoNTA during afternoon in diurnally active patients should be pursued in order to achieve treatment optimization. Acknowledging methodological differences, which might have prevented the results from being extrapolated, we found that the circadian time of BoNTA administration in CM patients does not appear to strongly influence either the response rates of treatment or its safety/tolerability profile, as demonstrated by comparable improvements between AM vs. PM-treated patients in all efficacy outcomes after three sessions or nine months of BoNTA exposure, compared to the pre-treatment period. Moreover, the safety/tolerability profile, as also satisfaction to treatment, was also similar between groups.

As mentioned earlier, methodological differences can possibly account for discrepancy between our results and those reported by Packard et al. [11] in relation to the impact of the circadian time of BoNTA administration on the efficacy outcomes [11]. In the latter setting, investigators compared 90 AM vs. PM patients with CM undergoing ≥the 4th cycle of BoNTA injection therapy, only if those were considered responders to BTA therapy defined as ≥30% reduction in headache days and/or headache intensity during three previous injection cycles. Participants were unequally allocated to treatment groups with 55 of them to be treated AM and 35 PM, and afterward they were mainly tested for differences in the mean number of headache days during 90 days post-BoNTA injection.

The study design and part of the methodology we applied were different to those of the two other available studies [11,12]. In this study, we have tested an equal number of consecutive diurnally active BoNTA-naïve CM patients scheduled to be treated with Botox^®^ 100 UI/fl, Allergan-Abbvie, Hellas in our Headache Clinics at morning or afternoon hours (50 vs. 50) for changes in efficacy outcomes after commencing three consecutive BoNTA sessions compared to baseline. Opposite to the Packard et al. study [11], we avoided to selectively include just responders to BoNTA, because we intended to seek differences in rates of both responders and non-responders to BoNTA and be able as such to fully explore the tested hypothesis. In addition, we compared AM vs. PM-treated patients for all clinically significant efficacy outcomes, including, apart from just changes in mean monthly headache days, differences in pain intensity, as well as the difference in days with consumption of acute headache medications from baseline to a 2-month period after the third repeated BoNTA administration. However, we should acknowledge that the retrospective design we applied is a limitation of our study, thus potentially making the present results not completely comparable to those previously reported. Nonetheless, provided that we have adequately clinically tested a larger sample size, compared to other studies, evenly allocated to study groups, we might suggest that we have been able to produce results that are statistically and clinically valid.

## 4. Conclusions

To conclude, we were not able to identify significant differences between patients treated in the AM vs. PM so as to demonstrate that the circadian time of administration should be considered to guide the therapeutic BoNTA protocol for each patient to optimize good and safe outcomes. Considering the highly speculative nature of explanatory hypotheses offered to support that PM-treated patients respond better to BoNTA, because of improved efficiency of cell entry and decreased toxin spread during afternoon administration to allow higher doses of BoNTA subtype-1 being injected [10], we strongly advocate in favor of the view that further research is needed to test the effect of the circadian time of BoNTA administration on its efficacy and safety/tolerability profile, focusing on chronopharmacokinetic analysis, mathematical modeling and biological markers of the timing system, before definite conclusions can be drawn on this issue.

## 5. Materials and Methods

This was a retrospective, multi-center study, conducted according to ethical principles of the Helsinki Declaration. Eligibility was confirmed by a protocol-specific checklist, as previously described [13,14] and written informed consent was obtained from each patient. Adult patients (>18 years) at enrollment were included in this study if they (i) had a definite clinical diagnosis of CM with or without MOH, (ii) were considered as non-responders to previous preventive oral medications, (iii) administration of such previous orally administered migraine preventives had to be discontinued at least 3 months prior to study entry, (iv) were scheduled to be treated with BoNTA, according to regulatory and clinical practice guidelines, (v) were diurnally active, (vi) had agreed to keep headache diaries as per their treating physicians’ instructions and (vii) were consistent in conducting the phone interviewing to assess safety/tolerability, according to the study needs. Patients with presence of shift work within the last 3 months of enrolment, those with history of either circadian rhythm sleep disorder or major psychiatric disorder, as well as those having been previously exposed to other injectable migraine prophylactics, were excluded throughout from the study.

BoNTA (Botox^®^ 100 UI/fl, Allergan-Abbvie, Hellas) was administered from certified BoNTA injectors throughout the study period at predefined cranial and cervical sites that align with the distribution for input to the trigeminal sensory system, including the procerus, corrugator, frontalis, temporalis and occipitalis muscles as well as the trapezius and paraspinal muscles. We used two BoNTA vials, while each 100 UI vial was diluted with 2 mL 0.9% sterile normal saline for a dilution of 5 UI/0.1 mL and was commenced to each patient at a fixed dose of 155 UI every 3 months, according to the PREEMPT paradigm [5] and our previously published experience [13,14]. In addition to the fixed 155 UI, up to 40 UI BoNTA were allowed to be administered in selected patients, according to the “follow the pain” approach, which may result in improved outcomes, compared with the fixed dose of 155 UI [24]. As such, BoNTA was commenced at a dose range between 155 and 195 UI.

In total, patients were scheduled to receive three treatment cycles in order to complete the study, and subsequently the study duration was 9 months. Participants were classified to those scheduled to be treated in the morning hours from 8:00 to 12:00 (AM; group I) or afternoon hours from 13:00 to 18:00 (PM; group II).

We mainly sought to identify potential differences in headache outcomes and tolerability of AM vs. PM-treated patients. Patients’ headache diaries and specific interviewing, retrieved from their medical files, were used to document changes in the efficacy variables during the treatment period. The primary headache outcome, which was assessed according to study groups, was the crude efficacy of BoNTA as expressed by the change in mean number of monthly headache days from baseline (T0–3 months period before the first BoNTA administration) to a 2-month period after its third administration (T3). Moreover, we looked at comparing other secondary headache outcomes according to study groups between T0 and T3, including the change in the number of days with peak moderate/severe headache intensity, and the change in days/month with consumption of any abortive headache medications. Finally, patients with at least a 50% decrease in headache days at T3 vs. T0 were characterized as BoNTA responders, while those with at least 75% reduction were rated as good responders and those with 100% reduction as excellent responders, as previously described [13]. The percentage of reduction (50, 75, 100%) in headache days was then compared, according to AM vs. PM study groups.

Concerning the safety and tolerability evaluation, patients were contacted by phone at day 14 following every BoNTA infusion and any AEs including discomfort, neck pain, muscle spasm, inflammation were recorded in their medical files, to be retrieved later and then compared between groups. PGIC was used to evaluate and compare between groups the overall patients’ self-perceived impact of disease management and satisfaction from treatment with BoNTA at T3. Briefly, PGIC is a 7-point patients’ reported outcome (PRO) questionnaire, where 1 reflects “no change” and 7 a “considerable improvement” [25]. A cut-off of PGIC ≥ 5 was set to define patients experiencing a “clinically meaningful benefit” with BoNTA treatment, according to the IMMPACT recommendations [26].

### Statistical Analysis

Descriptive statistics were generated for all variables, depending on the nature of the variable. Comparison of categorical data between patients treated in the AM vs. those treated in the PM was performed using the two-sided chi squared tests, while the independent samples *t*-test was used to reveal the differences between groups in continuous data. For within-group comparisons, the changes in mean headache outcome scores from T0 to T3 were evaluated with the use of the paired samples *t*-test. For between-group comparisons, the changes in mean headache outcome scores were evaluated by subtracting each patient’s baseline value from her/his last value, and were calculated by employing the independent sample *t*-tests. All tests were two-sided and significance level alpha was set at *p =* 0.05 or lower. The SPSS for Windows (release 27.0; SPSS Inc., Chicago, IL, USA) conducted the statistics.

## Figures and Tables

**Table 1:** Demographic and clinical characteristics of AM vs. PM BoNTA-treated participants.

*Variable*	*AM-Treated Patients*		*PM-Treated Patients*	
*Participants*	*n* = 50		*n* = 50	
*n* = 100	*N*	*%*	*N*	*%*
**Gender**				
Females	42	84	47	94
Males	8	16	3	6
**Age ± SD (range)**	42.7 ± 10.2 (21–60)		44.6 ± 9.3 (30–60)	
**Number of previously used preventative medications**				
Median value (range)	3 (1–7)		3 (1–7)	
**Years** **± SD (range) with chronic migraine**	10.1 ± 3.7 (6–16)		10.4 ± 3.6 (6–18)	
**Psychiatric comorbidities**	35	70	39	78
Anxiety disorder	13		14	
Depression	10		12	
Mixed anxiety and depression disorder	10		10	
Bipolar disorder	2		3	
**Medication overuse headache**				
Yes	33	66	36	72
No	17	34	14	28

## Data Availability

The data that support the findings of this study are available from the corresponding author upon reasonable request.
